# Quantitative assessment of specific carbonic anhydrase inhibitors effect on hypoxic cells using electrical impedance assays

**DOI:** 10.1080/14756366.2017.1355306

**Published:** 2017-08-07

**Authors:** Luciana Stanica, Mihaela Gheorghiu, Miruna Stan, Cristina Polonschii, Sorin David, Dumitru Bratu, Anca Dinischiotu, Claudiu T. Supuran, Eugen Gheorghiu

**Affiliations:** a International Centre of Biodynamics, Bucharest, Romania;; b Faculty of Biology, University of Bucharest, Bucharest, Romania;; c Department of Biochemistry and Molecular Biology, Faculty of Biology, University of Bucharest, Bucharest, Romania;; d Neurofarba Department, Sezione di Scienze Farmaceutiche, Università degli Studi di Firenze, Sesto Fiorentino (Firenze), Italy

**Keywords:** Electrical cell substrate sensing, HT29 cells, hypoxia, carbonic anhydrase IX inhibitors, cellular platform

## Abstract

Carbonic anhydrase IX (CA IX) is an important orchestrator of hypoxic tumour environment, associated with tumour progression, high incidence of metastasis and poor response to therapy. Due to its tumour specificity and involvement in associated pathological processes: tumourigenesis, angiogenesis, inhibiting CA IX enzymatic activity has become a valid therapeutic option. Dynamic cell-based biosensing platforms can complement cell-free and end-point analyses and supports the process of design and selection of potent and selective inhibitors. In this context, we assess the effectiveness of recently emerged CA IX inhibitors (sulphonamides and sulphocoumarins) and their antitumour potential using an electrical impedance spectroscopy biosensing platform. The analysis allows discriminating between the inhibitory capacities of the compounds and their inhibition mechanisms. Microscopy and biochemical assays complemented the analysis and validated impedance findings establishing a powerful biosensing tool for the evaluation of carbonic anhydrase inhibitors potency, effective for the screening and design of anticancer pharmacological agents.

## Introduction

Carbonic anhydrase (CA, EC 4.2.1.1) isoform IX (CA IX)^1^ is a zinc transmembrane metalloenzyme recognised as a key component of the hypoxic microenvironment characteristic to many types of solid tumours, where cells are deprived of oxygen due to rapid proliferation and change of metabolism from oxidative phosphorylation to glycolysis. CA IX is involved in processes like tumour proliferation and growth, cell adhesion and intercellular communication, migration, and, most importantly, pH regulation^2^. CA IX maintains the intracellular pH favourable for tumour cell survival and growth while contributing to the acidification of the tumour environment and promoting tumour metastasis^3^. To do so, CA IX regulates pH and ion balance by catalysing the interconversion of carbon dioxide (CO_2_) to bicarbonate anion (HCO_3_^−^) and protons (H^+^)^4^ with its extracellularly oriented active site, whereas through its proteoglycan-like domain and extracellular carboxyl terminal tail influences cell–cell adhesion. CA IX is one of the few tumour-associated CA isoenzymes known so far; it is highly overexpressed in a broad range of solid tumours (carcinoma of the cervix, kidney, breast, lung, head and neck and tumours of the brain)^5^ yet has only a very limited distribution in normal tissues (e.g. gastrointestinal tract – epithelia of the glandular stomach, small intestine and gallbladder)^6^. As such, CA IX has become a validated target for anticancer therapy via the design of specific inhibitors, one such compound, SLC-0111, being in phase I/II clinical trials for the treatment of advanced, metastatic solid tumours (see clinical trials.gov, NCT02215850).

Sulphonamides and sulphocoumarins are two wide classes of compounds able to inhibit the catalytic activity of CA IX by different mechanisms^7^^,^^8^, and have been proposed as potential antitumour therapeutic compounds. Often, the efficiency of these carbonic anhydrase inhibitors (CAIs) is determined based on a cell-free assessment of enzymatic activity and end-point biochemical cell analyses. In contrast, real-time, long-term analyses are lacking.

Aiming to further the understanding of the dynamics of interaction between CAIs and the target enzyme overexpressed in live cells under hypoxic conditions, we advance a multiparametric cell biosensing platform. In contrast to the end-point analyses, the use of label-free bioanalytical platforms allows dynamic evaluation of discrete, non-lethal effects of external stimuli at the cellular level and enable a quantitative, multiparametric assessment of potential bioeffects^9^^,^^10^. As such, they are invaluable tools for the development and preclinical testing of novel therapeutic compounds. To this end, electrical impedance spectroscopy (EIS), a non-invasive, label-free technique that allows real-time monitoring^11^ of dynamic cellular responses induced by external biological and chemical compounds^12^, is advanced for testing the effect of several inhibitors (sulphonamides and sulphocoumarins compounds) on HT29 tumour cells. The HT29 colorectal carcinoma cells were selected as a model cell line due to their known overexpression of CA IX under hypoxic conditions and ability to adapt to hypoxic conditions through the interplay between metabolic changes and hypoxia-induced signalling^13^.

We appraise the inhibitory capacities of selected inhibitory compounds and reveal the dynamics of the cellular processes that underlie hypoxia and CA IX inhibition, indicating anticancer drug screening as a reliable application of EIS. As model inhibitory compounds we selected: a fluorescent, high affinity CA IX inhibitor, CAI #1, a potent, yet less CA IX specific inhibitor, CAI #2 (proved to have moderate inhibitory capacity for cytosol and membrane bound CA isoforms II and IV), a positively charged, membrane-impermeant derivative that has been reported as a good inhibitor of CA IX and CA II, CAI #3, and a sulphocoumarin demonstrated to be an effective CA IX inhibitor, CAI #4. The clinically used inhibitor acetazolamide (AZA) that indiscriminately inhibits all CAs was used as a control. Complementary biochemical assays confirmed the EIS results, offering additional information regarding cell viability, redox status and actin cytoskeleton organisation as modulated by CAIs under hypoxic conditions.

Impedance studies with cells in hypoxic conditions are seldom reported^14^^,^^15^, with a focus on barrier function assays. To the best of our knowledge, our study demonstrates for the first time the dynamic evaluation of cellular effects induced by CAIs and indicates a powerful biosensing approach for the assessment of CAIs potency, effective for the screening and design of a wider range of anticancer pharmacological agents.

## Materials and methods

### Cell cultures

Human colon cancer HT29 cells, (ATCC HTB38), were grown in Dulbecco-modified Eagle medium supplemented with 10% foetal bovine serum (FBS) and penicillin-streptomycin (100 IU/ml–0.1 mg/ml) and kept at 37 °C in a 5% CO_2_ humidified incubator (MCO-20AIC Sanyo, Osaka, Japan). All culture media and supplements were purchased from Invitrogen™ (Carlsbad, CA) and Sigma-Aldrich (St. Louis, MO).

Cells were seeded at a concentration of 4.5 × 10^5^ cells/ml on IBIDI electric cell-substrate impedance sensing ( ECIS, IBIDI, GmbH, Germany) culture ware (8 W10E PET) and cultured for 2 d before experiments to reach confluence. Exposure to hypoxic (1% O_2_) and normoxic (21% O_2_) conditions together with specific treatment compounds was performed after the cells have reached confluence in standard growth conditions. Upon culture media exchange, the medium was aspirated off and replaced with either standard growth medium or medium containing CAIs (100 µm concentration^16^). Cells were further incubated for 12 h at 37 °C in the incubator/hypoxic chamber throughout the experiments.

### Hypoxic conditions

Exposure of cells to a hypoxic environment was achieved using a modular incubator chamber (MIC-101, Billups-Rothenberg Inc., San Diego, CA). Cells were placed inside the chamber flushed with a gas mixture containing 94% N_2_, 1% O_2_ and 5% CO_2_. Once gas equilibrated, the chamber was sealed and placed inside the incubator and cells were subjected to hypoxia for 12 h. Control cells were kept in normoxic conditions within the same humidified incubator, with 5% CO_2_. Both incubation units were equipped with electrode mounts for impedance evaluation under controlled conditions.

### Chemicals

Four types of recently proposed CA IX inhibitors were used in experiments: a fluorescent one CAI #1 (fluorescein-thioureido-homosulfanilamide)^17^, CAI #2 (1-N-(4-sulfamoylphenyl)-2,4,6-trimethylpyridinium perchlorate)^18^^,^^19^, CAI #3 (1-N-(4-sulfamoylphenylethyl)-2,4, 6-trimethylpyridinium perchloride) (19) and CAI #4 (6-(benzyloxy)-1,2-benzoxathiin-2,2-dioxide)^20^^,^^21^, while AZA (Sigma-Aldrich), a standard inhibitor of CAs was used as a control. The inhibitors were dissolved in PBS with 20% DMSO at 10 mm concentration and diluted in culture medium to the final concentration of 100 µm just before cell treatment. The structures of the inhibitors, developed at the University of Florence, Italy, are presented in the “Results” section. CAIs showed *K_i_* values in the (10th of) nm range as assessed by CO_2_ hydration methods using the purified CA domain of CA IX.

### Electrical impedance spectroscopy measurements

The EIS enables multi-parameter, real-time monitoring of the interaction between cells and substrate and the study of cellular and subcellular processes in response to external stimuli^10^^,^^22^. A 4294 A Precision Impedance Analyzer (Agilent, now Keysight Technologies, Santa Clara, CA) interfaced with in house multiplexing module for up to eight channels was used for recordings. An AC signal of 100 mV amplitude, zero DC bias, within 100 Hz–100 kHz frequency range (100 frequency points with logarithmic distribution) was applied and spectra were recorded at selected time intervals (every 5 min). Data were collected and processed using a custom developed LabView interface. The whole spectra of the complex impedance Z*(fr,t)=Re[Z(fr,t)]+i Im[Z(fr,t)] were analysed and complex fitted with a simplified equivalent circuit to derive time evolutions of specific circuit parameters as function of hypoxic conditions and CAIs’ effect. In view of a simplified biosensing tool, single frequency impedance analysis has been applied as well. The imaginary part of impedance at 10 kHz frequency allows direct evaluation of cell attachment and growth and was selected throughout the analysis. Impedance values were normalised using the formula [V(fr, t)-V(fr,0)]/V(fr,0) where V stands for the imaginary part of the complex impedance. Data analysis was realised using OriginPro 8.5 (OriginLab, Northampton, MA). All values were expressed as the mean ± standard deviation (SD). The statistical significance was assessed using OriginPro 8.5 (OriginLab), Student’s *t*-test, and a *p* values <.05 was considered statistically significant.

### Optical microscopy experiments

Epifluorescence has been used to evaluate the expression of CA IX in cells subjected to hypoxic conditions and treated with the fluorescent CAI #1. Moreover, complementary Differential Interference Contrast (DIC) and Bright Field Reflected Light (BFRL) assays have been used to assess cell morphology and cell-surface contacts. The microscopy set-up contained an AxioObzerver Z1 (Zeiss, Germany) microscope, a 40 × 0.95 NA objective (Zeiss, Jena, Germany), an ANDOR EMCCD camera, and an environmental control enclosure (CO_2_ and temperature, OKOLab, Pozzuoli, Italy). Cells were seeded at a concentration of 5 × 10^4^ cells/ml on Petri dishes with glass bottom (World Precision Instruments, Sarasota, FL) and used for experiments the next day.

#### Intracellular glutathione (GSH) detection and quantification

Intracellular glutathione was stained using CellTracker Green 5-chloromethylfluorescein diacetate (CMFDA; Molecular Probes, Invitrogen). The culture medium was removed and the cells were incubated in FBS-free culture medium with 5 µm CMFDA at 37 °C and 5% CO_2_ for 30 min. After washing with pre-warmed media, cells were incubated for another 30 min in FBS-free culture medium to allow the hydrolysis of CMFDA to the fluorescent 5-chloromethylfluorescein (CMF) by intracellular esterases and conjugation with GSH or the diffusion of the unconjugated dye. Images were acquired using an inverted fluorescence microscope (Olympus IX71, Tokyo, Japan). The entire cell area was outlined based on the phase contrast images and was transposed on the CMFDA staining images in order to quantify the fluorescence intensity of the reduced GSH-5-CMF (GSH-CMF) complex. The fluorescence was normalised to the corresponding area in order to obtain uniform results regardless of cell size. The quantification of GSH was performed using ImageJ software (NIH, Bethesda, MD) for 200 cells per experimental group, selected from 20 different fields from four independent experiments.

#### F-actin staining

Actin cytoskeleton morphology was imaged via fluorescence imaging using cells fixed with 4% paraformaldehyde for 20 min and permeabilised with 0.1% Triton X-100 – 2% bovine serum albumin solution (prepared in PBS) for 1 h at room temperature. Filamentous actin (F-actin) was labelled with 20 µg/ml phalloidin conjugated with fluorescein isothiocyanate (FITC; Sigma-Aldrich, Darmstadt, Germany) for 1 h at room temperature. Nuclei were stained with 2 µg/ml 4′,6-diamidino-2-phenylindole (DAPI; Invitrogen) for 10 min at room temperature. Images were acquired using an Olympus IX71 inverted fluorescence microscope.

#### Lysosome staining

Lysosomes were stained with 100 nm LysoTracker Green DND-26 (Molecular Probes, Invitrogen) for 30 min at 37 °C, followed by the counterstaining of nuclei for 10 min at room temperature with 2 µg/ml Hoechst 33342 (Molecular Probes, Invitrogen). After washing the cells with PBS, images were acquired using an Olympus IX71 inverted fluorescence microscope.

### Biochemical assays

#### MTT assay

Cell viability was measured using the 3-(4,5-dimethylthiazol-2-yl)-2,5-diphenyltetrazolium bromide (MTT; Sigma-Aldrich) assay based on the reduction of the tetrazolium salt to purple, insoluble formazan crystals by the NAD(P)H-dependent cellular oxidoreductase enzymes in the viable cells. The culture medium was removed and the cells were incubated with 1 ml of 1 mg/ml MTT solution for 2 h at 37 °C. The formazan crystals formed in the viable cells were then dissolved with 1 ml of 2-propanol (Sigma-Aldrich). Absorbance was measured at 595 nm using a microplate reader (GENios Tecan, Austria).

#### Preparation of cell lysates

HT29 cells were harvested from culture flasks at the end of hypoxia and washed with PBS. Cells were lysed by sonication (30 s for three times) on ice with an ultrasonic processor (Hielscher UP50H, Germany) and centrifuged at 10,000 × *g* for 10 min at 4 °C. Supernatants were collected for lipid peroxidation assay and Western blot. The protein concentration was determined using the Bradford Reagent (Sigma-Aldrich) and bovine serum albumin as the standard protein.

#### Lipid peroxidation assay

Malondialdehyde (MDA) level was assessed as a marker of lipid peroxidation using a fluorimetric method^23^. A volume of 200 µl cell lysate was mixed with 700 µl of 0.1 N HCl. After 20 min at room temperature, 900 µl of 0.025 M thiobarbituric acid were added and the mixture was incubated for 65 min at 37 °C. The MDA concentrations, nmol/mg protein, were obtained using fluorescence recordings (JASCO Spectrofluorometer FP-6300, *λ*_ex_ = 520 nm; *λ*_em_ = 549 nm) and 1,1,3,3-tetramethoxypropane as standard.

#### Western blotting

Cell lysates corresponding to 40 µg of protein were separated on 10% sodium dodecyl sulphate-polyacrylamide gel electrophoresis under reducing conditions and transferred onto 0.4 µm poly(vinylidene difluoride) membrane (Millipore, Billerica, MA) in a wet transfer system (Bio-Rad, Hercules, CA). The membranes were blocked with blocking solution included in the WesternBreeze Chromogenic kit (Invitrogen) for 30 min at room temperature. Primary mouse polyclonal antibodies anti-human p53 and anti-β-actin (dilution 1:250; Santa Cruz Biotechnology, Santa Cruz, CA) were used for protein detection. The membranes were processed in accordance with the manufacturer’s instructions, using anti-mouse secondary antibodies coupled with alkaline phosphatase and 5-bromo-4-chloro-30-indolylphosphate/nitroblue tetrazolium as the chromogenic substrate. The resulting bands were imaged with a ChemiDoc MP system (Bio-Rad) and quantified using Image Lab software (Bio-Rad).

### Statistical analysis

All results were expressed as the mean ± SD of five independent experiments and were represented as a percentage relative to control. The statistical significance was assessed using GraphPad Prism software (La Jolla, CA), one-way ANOVA test followed by a *post-hoc* Bonferroni test. A *p* values <.05 was considered statistically significant.

## Results

### Cellular dynamics under hypoxic conditions revealed by EIS measurements

**Figure 1.**
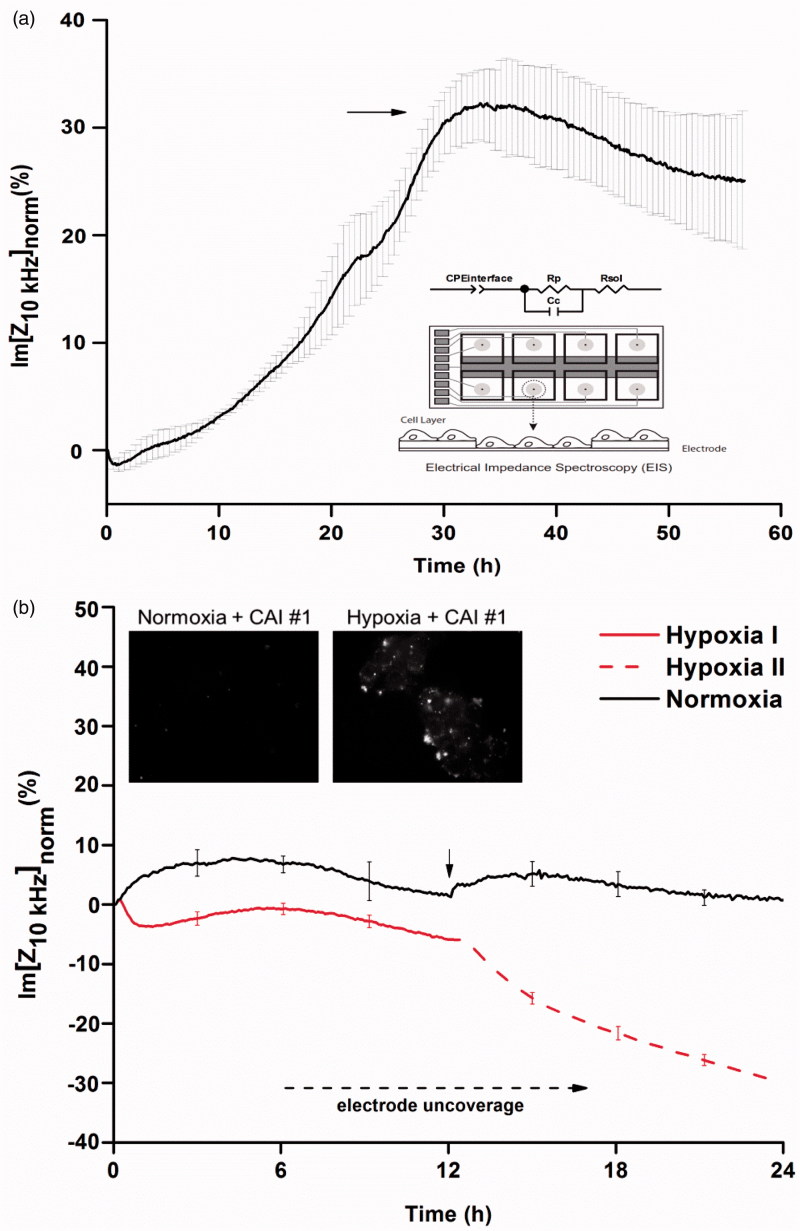
(a) The normalised impedance values (imaginary part at 10 kHz) increase as cells adhere and spread on the electrodes until a monolayer is formed. Data are represented as mean ± SD for *n* = 7. The electrode set-up and the simplified equivalent circuit for assessing EIS data on cell monolayers are presented in the inset. (b) The response of cells to hypoxic conditions, two subsequent hypoxia phases- impedance data (the imaginary part at 10 kHz). Data are represented as mean ± SD for *n* = 6. Inset CA IX overexpression highlighted through fluorescent CAI #1 binding.

EIS measurements of cells in the frequency range of 100 Hz–100 kHz were performed to monitor cell growth and to evaluate in real-time and non-invasively the cell monolayer response to hypoxia and CAIs effects. Depending on the number, morphology, degree of attachment and electrical properties, interfacial effects and membrane processes of the cells attached to the electrodes shape the frequency dependence of system’s impedance. An equivalent circuit, presented in inset Figure 1(a), comprising a constant phase element (CPE) accounting for the cell monolayer-electrode interface, a resistor (Rp) that defines the paracellular route in parallel with a capacitor (Cc) which describes the cellular monolayer and a resistor for the bulk solution (Rsol) is suitable for fitting the complex impedance spectra in the 100 Hz–100 kHz domain. Analysis of the impedance spectra in respect to the equivalent circuit is presented in Supporting information. Rp and CPE values were demonstrated to be the best reporters of cellular state and electrode coverage and hence suitable for cell assessment. Alternatively, impedance values at single frequencies can be used for monitoring system’s response. The 10 kHz frequency is suitable^24^ for monitoring the adhesion of cells onto electrodes, provides information regarding the membrane impedance and was found to be optimal for the particular electrode configuration (i.e. 10 circular 250 μm diameter active electrodes connected in parallel on a common gold pad) of the culture ware used in the experiments. At this frequency, the time evolution of the impedance (in particular, the imaginary part of impedance) is able to show reliably changes of cell monolayer integrity and of cell-support interface for both cell growth and in response to hypoxic conditions (Figure 1(a,b)).

Upon cellular confluence, achieved when impedance values reached a plateau (Figure 1(a), arrow), the cells were exposed to hypoxic conditions. In response to a first (12 h) phase of hypoxia, the impedance values have a distinct evolution from the one corresponding to normal growth conditions. Cellular effects due to pH changes determined by the onset of hypoxic conditions shape the dynamics of the values of impedance during the first hours of hypoxia. Tumour cells (HT29 including) were demonstrated^25^ to overexpress in hypoxic conditions CA IX and CA XII, transmembrane isoforms of CA generally considered key pH regulators^26^. The impedance data reveal a transient drop in normalised impedance values consistent with cellular uncoupling at the surface of the electrodes and progressive expression of CA IX.

As expected, the overexpression of CA IX induced by hypoxic conditions is able to promote tumour cell survival as well as tumour-specific pH homeostasis. This fact is reflected in the subsequent increase in impedance values (Figure 1(b) Hypoxia I up to first 6 h) and is confirmed based on fluorescent CA IX inhibitor binding (inset). Increased cell mobility and changes in cellular status (including cell death) are evident (decrease of the values in the following 6 h) throughout the hypoxic exposure, yet cells are still able to maintain their cell-surface contacts that enable adequate impedance assessment. Notably, HT29 cells ability to adapt to hypoxic conditions through the interplay between metabolic changes and hypoxia-induced signalling^13^ is revealed by impedance assays during these first 12 h of hypoxia.

Furthermore, during the second phase of hypoxia (another 12 h) the impedance values decrease abruptly suggesting a looser contact with the surface and a disturbed cellular state upon repeated/prolonged hypoxic stress.

Biochemical assays performed after the first and the second phase of hypoxia confirmed impedance findings offering additional information regarding viability, oxidative stress and p53 levels. The results, presented in Supporting information (Figure S1) revealed a stronger effect of the second stage of hypoxia in comparison to cells kept in hypoxic conditions for only 12 h. Cellular responses to hypoxia include metabolic changes induced by the lack of oxygen and an increased generation of reactive oxygen species (ROS) which attack preferentially the membrane lipids. The prolonged hypoxia induces oxidative injury to unsaturated fatty acids through ROS, resulting in compromised lipid membrane matrix dynamics, altered cell morphology with important effects on the actin cytoskeleton and actin-dependent cellular processes, and decreased cell viability. The tumour suppressor p53 is sensitive to hypoxia-mediated oxidative stress^27^ and its activation starts the process leading to apoptosis if the DNA damage cannot be repaired.

As such, a decrease in cell viability down to 85% ±5 for the first phase of hypoxia and 62% ±2 for the second phase in comparison to control is accompanied by specific increases in MDA and p53 levels (Supporting information Figure S1). The increase in MDA levels indicates^28^ an enhanced lipid peroxidation and indirectly, the generation of ROS. The activation of p53-induced apoptosis^29^ is revealed by a two-fold increase in expression of this protein for the second stage of hypoxia.

Moreover, the effect of hypoxia on cell morphology and adhesion has been confirmed based on DIC and BFRL (Supporting information Figure S2A) and is highlighted by an altered organisation of the actin cytoskeleton (Supporting information Figure S2B).

Since functional CAIs would determine an augmentation of the hypoxic conditions effect at the cellular level, the evaluation of the two hypoxia steps was a prerequisite for establishing the suitable “sensitivity window” for our assay. The extent of the cellular effects of the second phase of hypoxia is significant, hindering detailed electro-optic assessment; therefore, to increase the sensitivity of the assay, the effect of CAIs was further interrogated only for cells exposed to the first phase (the first 12 h) of hypoxia.

### Characterisation of the inhibitory capacity revealed by EIS measurements

To test the capability of EIS assays to reveal the inhibitory potency, five model inhibitory compounds among sulphonamides and sulfocoumarins^21^^,^^30^^,^^31^ (Figure 2(a)) were chosen.

Figure 2.(a) Chemical structures of the CA IX inhibitors used in this study. (b) The dynamics of normalised impedance data during 12 h of hypoxia as a function of model CAIs exposure (100 μm). Data are represented as mean ± SD for *n* = 6. (c) The dynamics of a characteristic equivalent circuit component derived from the complex fitting of impedance data during 12 h of hypoxia as a function of model CAIs exposure (100 μm). (d) Characteristic impedance changes at a selected time point (after 6 h of treatment) revealing the CAIX inhibitors potency on HT29 cells under normoxic and hypoxic conditions. Data are represented as mean ± SD for *n* = 6. Statistical significance *p < .05, **p < .01 and ***p < .001 versus hypoxic conditions based on paired *t*-test. Inset: Statistical significance of impedance data for discrimination between CAI’s potency *p < .05, **p < .01 and ***p < .001.

**Figure:**
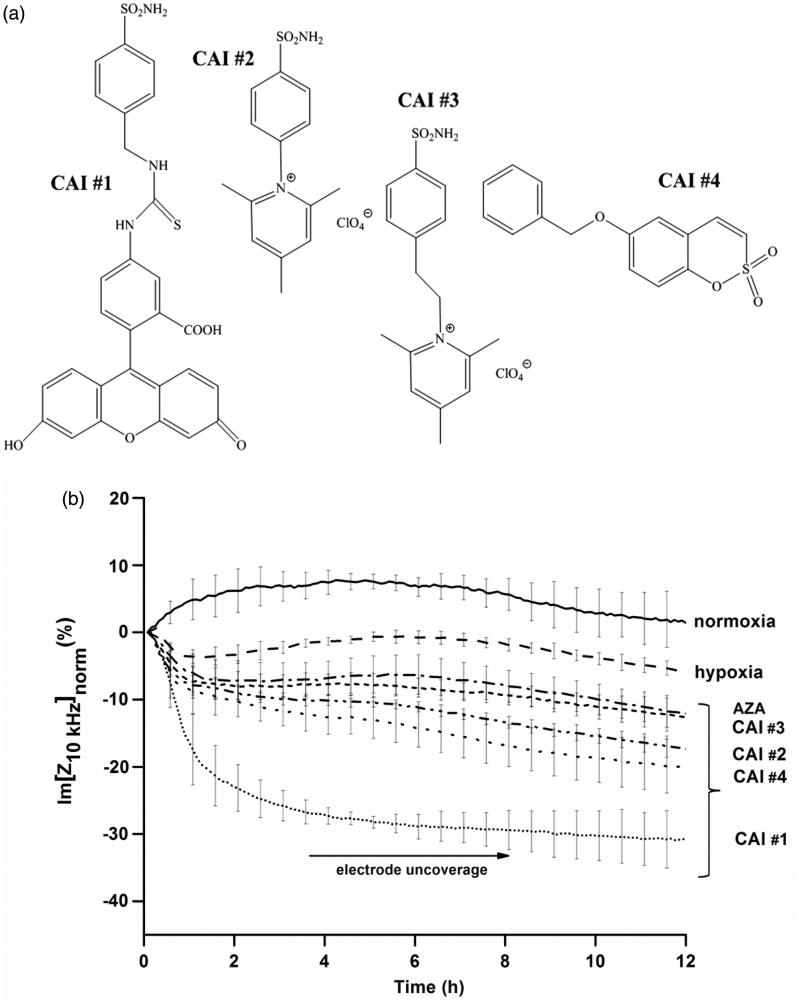

**Figure:**
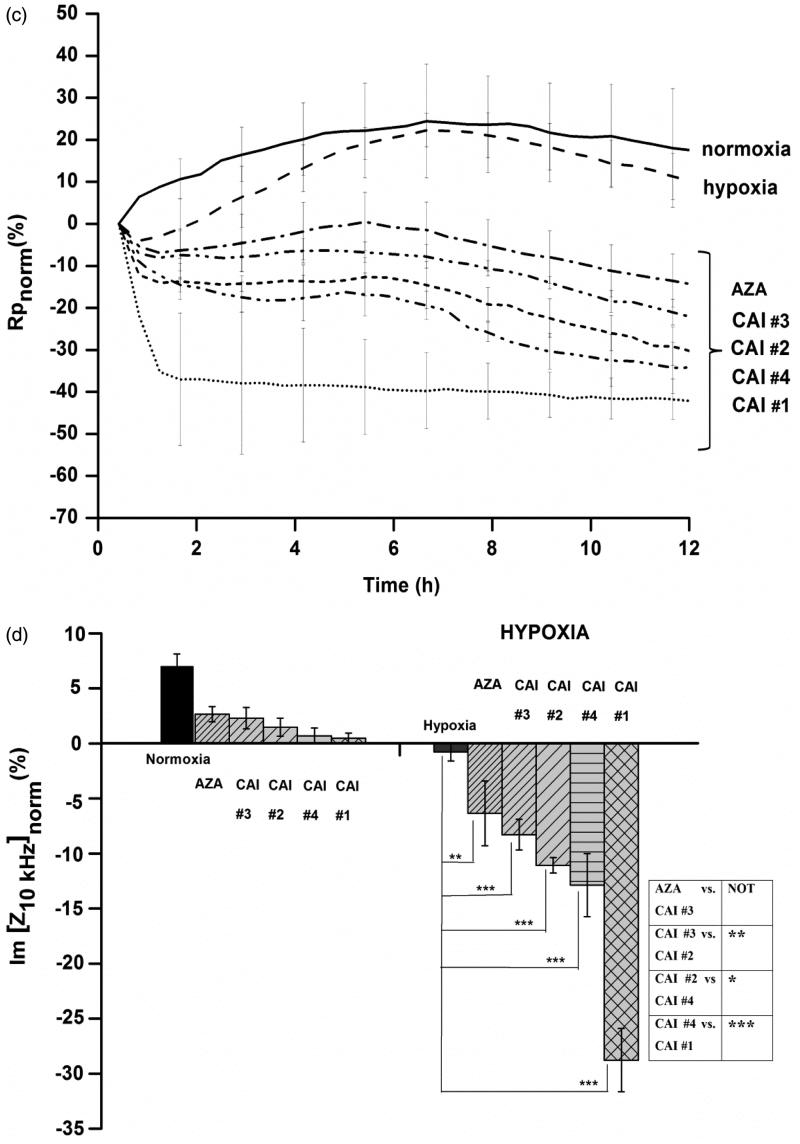

Sulphonamides are a group of inhibitors that target efficiently CA IX^32^ and their mechanism of inhibition is well-understood^26^. Among the sulphonamides model inhibitory compounds used in the analysis, CAI #1 is fluorescent and has a high affinity for CA IX^18^, CAI #2 has moderate inhibitory capacity in the case of CA II and IV^30^^,^^31^ (cytosol and membrane-bound respectively), CAI #3 is a positively charged, membrane-impermeant derivative that has been reported as an inhibitor of CA IX and, intriguingly, of CA II^33^. CAI #4 is a 6-substituted sulphocoumarin reported as an effective CA IX inhibitor^8^. The clinically used inhibitor AZA which indiscriminately inhibits all CAs (16 mammalian CA isoforms identified)^34^ and has been extensively used in CA inhibition studies, was considered herein a control.

Although HT29 cells show intermediate CA IX expression under normoxic conditions, literature reports^35^ document that only during hypoxia CA IX becomes catalytically active and able to bind sulphonamides^35^. In this context, CA IX expression in cells subjected to normoxic and hypoxic conditions was evaluated by epifluorescence assay based on the incubation of cells with the fluorescent CAI#1. A fluorescent signal was detected only in hypoxic cells, being absent in the normoxic cells (Figure 1(b) inset and Supporting information Figure S2A). The lack of the fluorescence signal in the cells kept in normoxic conditions indicates CAI #1 selectivity for CA IX expressed during hypoxia.

To monitor the effect of CAIs in conjunction with the hypoxic conditioning of cells (i.e. overexpression of CA IX) we have compared, using EIS, the behaviour of the cells incubated with CAIs in normoxic versus hypoxic conditions during 12 h of hypoxia (Figure 2(b)). The cells treated with CAIs and kept in normoxic conditions did not suffer significant changes as compared to control normoxic conditions but had highly different dynamics from the ones in hypoxic conditions.

CAIs application revealed a characteristic time evolution of the impedance value (the imaginary part at 10 kHz) in comparison to normoxia and hypoxia alone. The pronounced decrease (Figure 2(b)) of impedance values in hypoxic conditions as a function of applied CAI, confirms an augmentation of the deleterious effects at the cellular level of the hypoxic conditions, due to impaired tumour pH homeostasis upon CA inhibition. Each inhibitor presented a characteristic behaviour indicating a different inhibitory capacity. As such, the cellular impact of CA inhibition is dependent on the type of CAI and can be used to assess the inhibition potency: for CAI #1 the drop in impedance had fast kinetics and the decreased value was the biggest, corresponding to extensive electrode uncovering. For CAI #4 and CAI #2, although the kinetics is fast at the beginning, the inhibitory capacity is lower in comparison to that of CAI #1 as demonstrated by an incomplete electrode uncoverage. Both CAI #3 and AZA seem to have the same, low inhibitory capacity.

Complex fitting of the impedance spectra (100 Hz–100 kHz) with the equivalent circuit presented in inset Figure 1(a) generates specific dynamics of the characteristic equivalent circuit components during 12 h of hypoxia as a function of model CAIs exposure. Consistent with effects on cell monolayer integrity as a function of CAIs inhibitory potency, the derived Rp values (Figure 2(c)), corresponding to the tightness of the cell monolayer, confirm the characteristic decrease for efficient CA inhibition also highlighted by the dynamics of the Im[Z] at 10 kHz. The characteristic dynamics of the other circuit parameters are provided in Supporting information (Figures S3–S6) and confirm the effects of CAIs on cell attachment (CPE) and cell membrane (Cc).

For biosensing purposes, the evaluation of impedance changes after 6 h of combined exposure (hypoxia and inhibitory compound) enables clear discrimination of CAIs potency as revealed in Figure 2(d). The high statistical significance is obtained versus both hypoxia and between specific CAIs (inset Figure 2(d)).

In conjunction with Supporting information (Figures S3–S6) data, these results indicate monolayer disruption, cell detachment from the substrate and subsequent cell death, as CA IX is being inhibited and the cells are no longer able to adapt to a hypoxic environment and, as such are important quantitative predictors of inhibitory potency.

Endpoint biochemical assays complement impedance measurements to highlight the effect of CAIs on cell viability, redox status and actin cytoskeleton organisation. Cell viability decreases for cells incubated with CAIs during hypoxia in comparison to cells kept only in hypoxic conditions (Figure 3(a)). This decrease is dependent on the type of CAI used and is well correlated to inhibitor potency. The lowest viability was recorded for cells undergoing combined hypoxia and CAI #1 treatment confirming CAI #1 as having the highest inhibitory potential.

Figure 3.CAIs effect on (a) cell viability, (b) intracellular GSH level, (c) accumulation of lysosomes (green: lysosomes labelled with LysoTracker Green DND26; blue: nuclei labelled with Hoechst 33342) (d) Quantification of lysosomes content based on fluorescence images, and (e) F-actin cytoskeleton organisation (green: F-actin labelled with phalloidin-FITC; red: F-actin labelled with Alexa Fluor 546-phalloidin; blue: nuclei stained with DAPI). Note the disappearance of cortical ring-shaped actin (solid arrows) and the punctuate staining of actin (arrow head). Scale bar, 20 µm. The results are represented as a percentage of normoxia (control), considered 100%. Data are expressed as mean ± SD for *n* = 6; ***p < .001, **p < .01 and *p < .05 versus hypoxic conditions.

**Figure:**
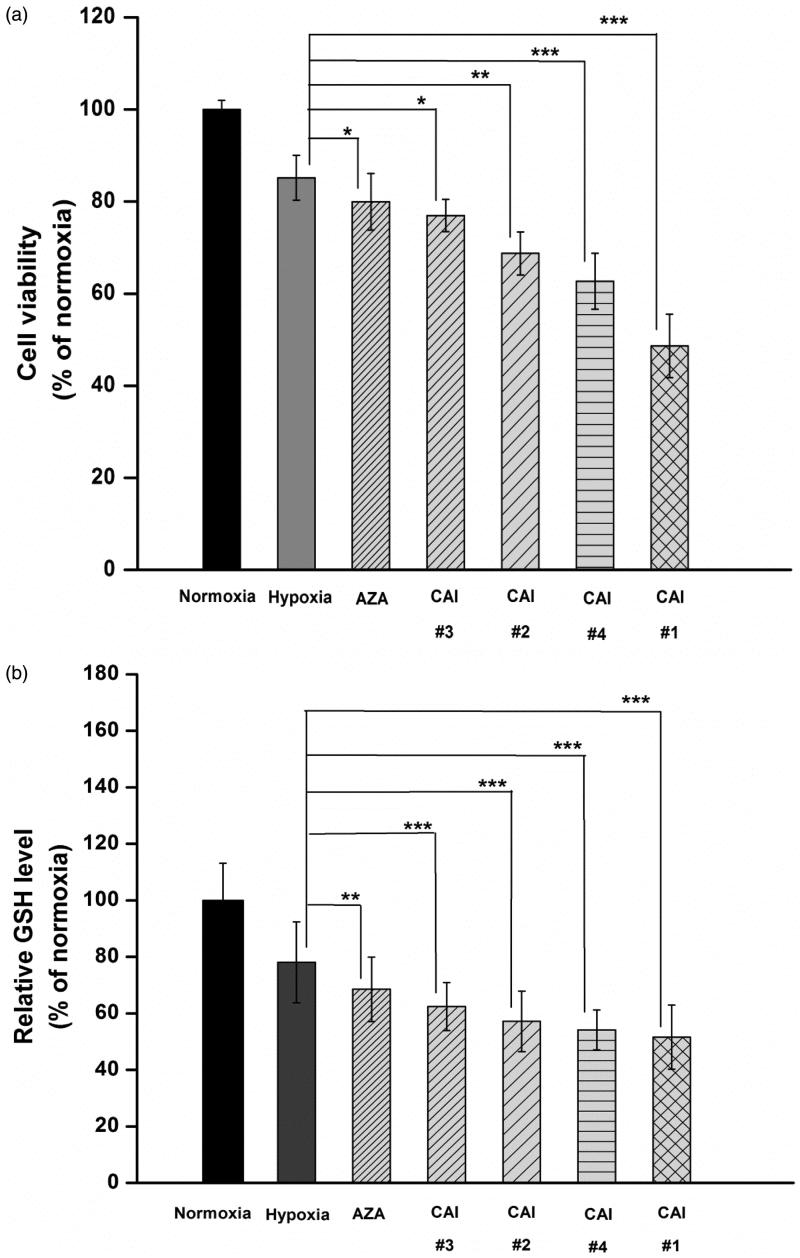

**Figure:**
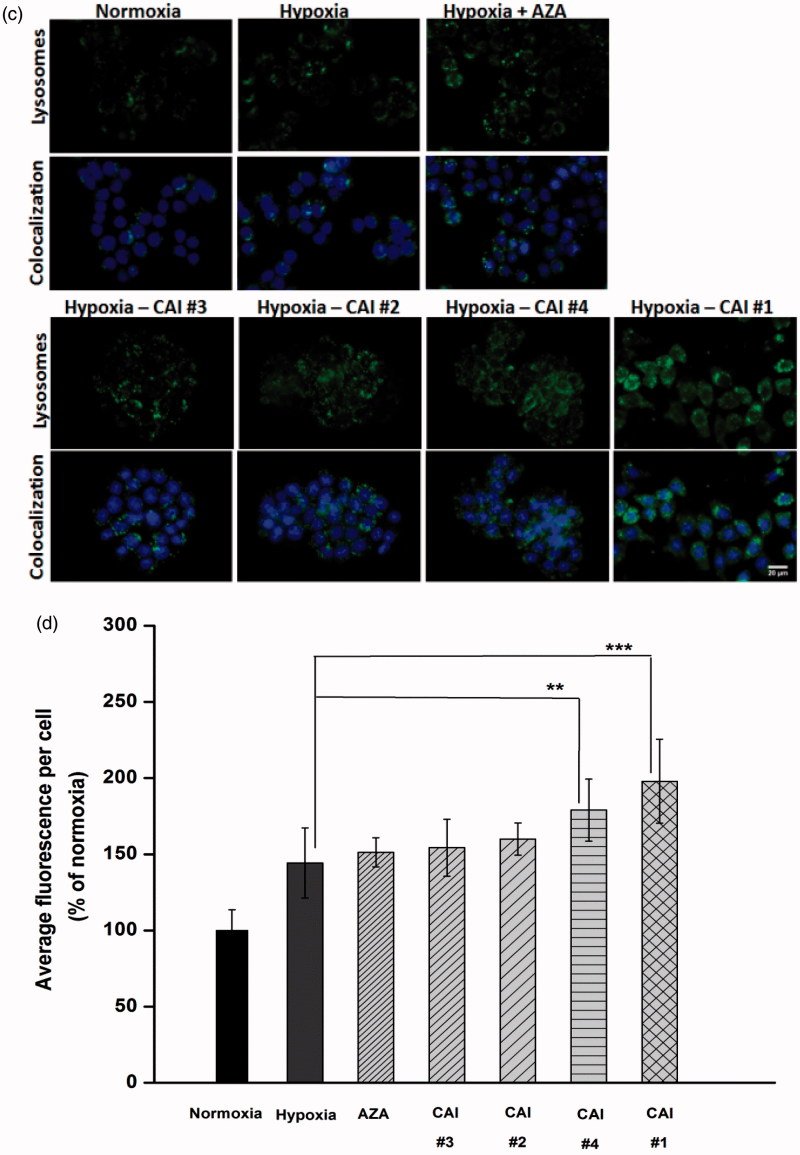

**Figure:**
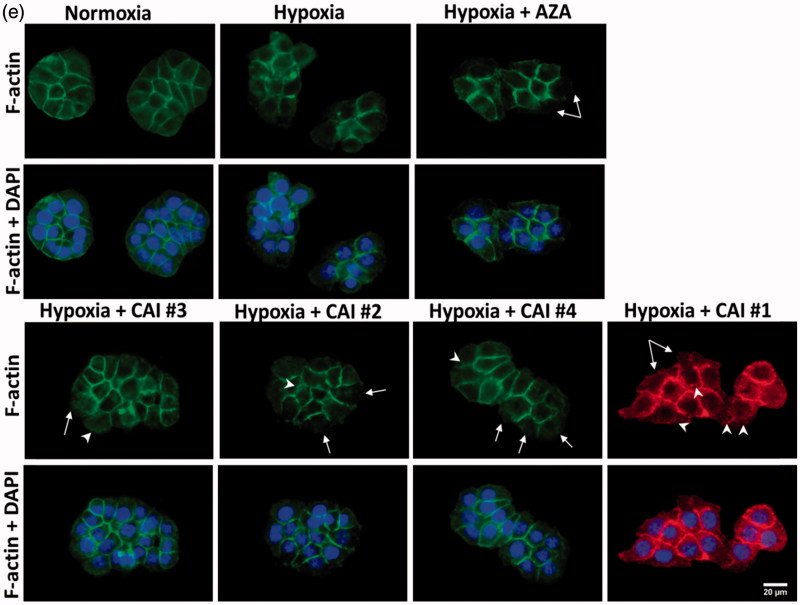

A decreased capacity of cells to maintain pH homeostasis under combined hypoxia and CAIs treatment is further confirmed based on the evaluation of intracellular GSH content (Figure 3(b)). Intracellular GSH concentration provides information regarding the induction and progression of oxidative stress and has been associated^36^ with the mitochondrial generation of ROS. In the process of oxidative stress alleviation, GSH acts as a barrier against excessive oxidation of redox-sensitive molecules^37^ and a lower intracellular GSH content suggests an altered redox status. The reduction in intracellular GSH level of HT29 cells incubated with CAIs during hypoxic conditions compared to that of cells subjected only to hypoxia (Figure 3(b)) follows the trend of viability data, indicating the induction of oxidative stress and depletion of GSH in all cellular compartments.

Lysosomes are important cellular organelles involved in the degradation of macromolecules and in the induction of apoptosis and autophagy. Previous studies have shown that hypoxia induces lysosomal autophagy and mitochondrial dysfunction in a manner dependent on expression of hypoxia inducible factor^38^^,^^39^. Therefore, to complete the picture of CAIs effect on hypoxic cells, we have evaluated their accumulation. In accordance with the decreased GSH level (Figure 3(b)), an accumulation of lysosomes was noticed after the incubation of HT29 cells with CAIs (Figure 3(c,d)) suggesting an increasingly oxidised environment. The accumulation of lysosomes in the case of cells incubated with CAI #1 was higher than for those incubated with the other CAIs indicating that more persistent damaged molecules have undertaken the degradation pathway.

The organisation of actin cytoskeleton filaments in HT29 cells kept in hypoxic conditions was also altered after CA IX inhibition. The presence of cortical ring-shaped actin observed in the cells grown in normoxia and simple hypoxia was significantly reduced (Figure 3(e) – solid arrows) as a result of the enhanced cellular stress induced by hypoxic conditions and CA IX inhibition. This feature was observed along with a diffuse pattern of actin staining (organisation of actin in the form of filaments was disrupted), the punctuate staining displayed being a characteristic of toxic effects on the cytoskeleton (Figure 3(e) – arrow heads).

## Discussion

Hypoxic environments are characteristic for a fast growing and/or solid tumours, being associated with aggressive tumour proliferation, resistance to therapy and a poor prognosis. CA IX is considered an endogenous marker of hypoxia, coordinating the carefully orchestrated tumour cell response to hypoxia that provides cancerous cells both a metabolic advantage as well as the capability to metastasise. This role in tumour progression and high incidence of metastasis is due to its capacity to maintain tumour intracellular pH homeostasis while acidifying the extracellular environment. Another important aspect of CA IX functionality, the potential to decrease cell–cell adhesion by destabilisation of E-cadherin links to the cytoskeleton, may contribute to the acquisition of increased tumour aggressiveness^6^. Therefore, CA IX is regarded as a clinically relevant, validated target for anticancer treatment as well as hypoxic tumour imaging.

Sulphonamides inhibit the catalytic activity of CA by binding to the zinc ion from the CA active site, whereas sulphocoumarins possess a diverse inhibition mechanism: they are hydrolysed within the enzyme active site to sulphonic acids which thereafter anchor to the water molecule bound to the zinc ion^34^. When cells grow in hypoxic conditions^34^, sulphonamide inhibitors are able to reduce the medium acidity by inhibiting the catalytic activity of CA IX enzyme, and thus the generation of hydrogen ions. Generically named CAIs, these compounds have been proposed as novel therapeutic/diagnostic agents^34^^,^^40^. Sulphocoumarins are among the latest CAI class introduced and have been shown to be effective CA inhibitors as well^8^^,^^41–43^.

The efficiency of CAIs is often predicted based on enzymatic activity and end-point biochemical cell analyses, yet the real-time, continuous assessment of cellular responses brings new opportunities to assist in the design of powerful, isoform selective inhibitors.

To this end, by electrically monitoring the dynamic changes of cellular responses induced by hypoxia and CA(IX)Is, we hereby show the capability of the EIS cell substrate sensing to assess the potency of anticancer drugs in hypoxic conditions. This study expands EIS proficiency as a label-free, real-time and non-invasive method to appraise a variety of cellular processes e.g. cell differentiation, cell spreading, cell motility, toxicology, cytotoxicity, cell cycle progression^12^^,^^44^ providing a quantitative analytical tool for evaluation of anticancer drug efficacy.

Single frequency impedance representation provides a straightforward biosensing tool. With appropriate frequency tuning (supported by analysis of impedance spectra using an equivalent circuit), the impedance data revealed changes at cell–cell and cell-surface level induced by the application of CAIs and allowed the differentiation between the inhibitory capacities of the compounds investigated. The interfacial capacitance, revealed by the imaginary part of impedance at a single frequency (10 kHz) provides a straightforward indicator of the overall cell-substrate adhesion. The results showed cell monolayer effects (including cellular detachment from the surface) as a function of effective and CA IX-selective inhibition. As such, cell adhesion and cytoskeletal activity changes, which are valuable indicators for understanding the cancer cell behaviour (such as proliferation, migration and invasion processes)^24^^,^^45^^,^^46^ were highlighted in the context of cell response profiles and cellular dynamics alterations as a result of the interaction of CAI compounds with cells. The different inhibitory capacities of the compounds could also be evaluated: CAIs #1 stands out as having a more potent effect than CAIS #4, #2, #3 and AZA, the least potent one. Complementary end-point cell viability assay indicated that indeed CAIs could successfully suppress the adaptive mechanism of hypoxic HT29 cells towards maintaining cell proliferation, promoting cell detachment and cellular death instead. As confirmed through complementary biochemical data, the exposure of cells with functionally altered CA IX (i.e. non-hypoxic HT29 cells or cells grown in the presence of potent inhibitors) to hypoxic conditions determines, in the context of altered pH homeostasis, the induction of cellular effects characteristic to oxidative stress: low GSH levels, accumulation of lysosomes and increase of their function, which may subsequently lead to the initiation of autophagy and cellular death.

Evolutions of the equivalent circuit parameters presented in Supporting information (Figures S3–S6) complement the one of trans-epithelial resistance Rp in Figure 2(c) highlighting subtle differences between the tested compounds in the mechanism of CA inhibition and possibly in the effects on the intracellular structures. The evolutions of CPE__T_ values, indicating cell-electrode interface, are clustered for hypoxia and low-efficiency inhibitors (AZA and CAI #3) and CAI #2 and #4 respectively, yet the biphasic evolution for CAI #4 is notable: there is an initial increase of the CPE__T_ values, that could correspond to a transient tightening of cell attachment for sulphocoumarin binding. These differences may, in fact, reflect the different inhibition mechanisms of the sulphonamides (zinc-binding inhibitors) on one hand and of the sulphocoumarins (prodrug inhibitors, hydrolysed first and then anchoring to the zinc-coordinated water molecule) on the other^34^.

Rearrangements of actin filaments and stress fibres formation are dynamic adaptive changes induced by hypoxia, in particular through the activity of Rho-GTPases^47^. We noticed the formation of stress fibres and the disruption of actin filaments when cells were treated with CAIs which could further have important consequences for cell morphology, adhesion and migration, changes highlighted by impedance data. Particular alterations of the cell membrane revealed by dynamics of the Cc element of the equivalent circuit could be used as additional indicators of CAIs antitumoural efficiency. Therefore, these results pinpoint once more the capability of the electrical impedance technique to sensitively assess cellular dynamics in conjunction with *in vitro* assays. In association with end-point biochemical data, the evaluation based on EIS and equivalent circuit analysis sheds light on the complex cellular effects determined by specific inhibitors and hypoxic conditions and stands out as an important biosensing tool.

## Conclusions

This study bridges the gap between classic end-point analyses and dynamic cell assessment and highlights process parameters relevant for assessing CA inhibition effectiveness. Potent CAIs are able to augment cellular changes characteristic for hypoxia progression: oxidative injury and compromised lipid membrane matrix dynamics, ROS accumulation, altered cell morphology with important effects on the actin cytoskeleton and actin-dependent cellular processes and decreased cell viability. The study demonstrates exquisite sensitivity of electric cell substrate impedance for the evaluation of CAIs potency towards a biosensing platform for novel and promising anticancer drugs development with clinical applications. Both the continuous cell dynamics and early time point (6 h) assessment were demonstrated to enable quantitative assessment of specific CAIs effect on live hypoxic cells.

## Supplementary Material

IENZ_1355306_Supplemental_Material.pdf

## References

[1] McDonald PC, Winum JY, Supuran CT, Dedhar S. Recent developments in targeting carbonic anhydrase IX for cancer therapeutics. Oncotarget 2012;3:84–97. 22289741 10.18632/oncotarget.422 PMC3292895

[2] Neri D, Supuran CT. Interfering with pH regulation in tumours as a therapeutic strategy. Nat Rev Drug Discov 2011;10:767–77. 21921921 10.1038/nrd3554

[3] Lou Y, McDonald PC, Oloumi A, et al Targeting tumor hypoxia: suppression of breast tumor growth and metastasis by novel carbonic anhydrase IX inhibitors. Cancer Res 2011;71:3364–76. 21415165 10.1158/0008-5472.CAN-10-4261

[4] Supuran CT. Advances in structure-based drug discovery of carbonic anhydrase inhibitors. Expert Opin Drug Discov 2017;12:61–88. 27783541 10.1080/17460441.2017.1253677

[5] Supuran CT. Structure and function of carbonic anhydrases. Biochem J 2016;473:2023–32. 27407171 10.1042/BCJ20160115

[6] Pastorekova S, Parkkila S, Parkkila AK, et al Carbonic anhydrase IX, MN/CA IX: analysis of stomach complementary DNA sequence and expression in human and rat alimentary tracts. Gastroenterology 1997;112:398–408. 9024293 10.1053/gast.1997.v112.pm9024293

[7] Supuran CT. How many carbonic anhydrase inhibition mechanisms exist? J Enzyme Inhib Med Chem 2016;31:345–60. 26619898 10.3109/14756366.2015.1122001

[8] Tars K, Vullo D, Kazaks A, et al Sulfocoumarins (1,2-benzoxathiine-2,2-dioxides): a class of potent and isoform-selective inhibitors of tumor-associated carbonic anhydrases. J Med Chem 2013;56:293–300. 23241068 10.1021/jm301625s

[9] Gheorghiu M, Enciu AM, Popescu BO, Gheorghiu E. Functional and molecular characterization of the effect of amyloid-beta42 on an *in vitro* epithelial barrier model. J Alzheimers Dis 2014;38:787–98. 24072066 10.3233/JAD-122374

[10] Gheorghiu M, David S, Polonschii C, et al Label free sensing platform for amyloid fibrils effect on living cells. Biosens Bioelectron 2014;52:89–97. 24035851 10.1016/j.bios.2013.08.028

[11] Gheorghiu E. Characterizing cellular systems by means of dielectric spectroscopy. Bioelectromagnetics 1996;17:475–82. 8986365 10.1002/(SICI)1521-186X(1996)17:6<475::AID-BEM7>3.0.CO;2-0

[12] Wang J, Wu C, Hu N, et al Microfabricated electrochemical cell-based biosensors for analysis of living cells *in vitro*. Biosensors 2012;2:127–70. 25585708 10.3390/bios2020127 PMC4263572

[13] Eales KL, Hollinshead KER, Tennant DA. Hypoxia and metabolic adaptation of cancer cells. Oncogenesis 2016;5:e190. 26807645 10.1038/oncsis.2015.50 PMC4728679

[14] Chanakira A, Kir D, Barke RA, et al Hypoxia differentially regulates arterial and venous smooth muscle cell migration. PLoS One 2015;10:e0138587. 26381529 10.1371/journal.pone.0138587 PMC4575051

[15] Kangwantas K, Pinteaux E, Penny J. The extracellular matrix protein laminin-10 promotes blood-brain barrier repair after hypoxia and inflammation *in vitro*. J Neuroinflammation 2016;13:25. 26832174 10.1186/s12974-016-0495-9 PMC4736307

[16] Dubois L, Peeters S, Lieuwes NG, et al Specific inhibition of carbonic anhydrase IX activity enhances the *in vivo* therapeutic effect of tumor irradiation. Radiother Oncol 2011;99:424–31. 21676479 10.1016/j.radonc.2011.05.045

[17] Cecchi A, Hulikova A, Pastorek J, et al Carbonic anhydrase inhibitors. Design of fluorescent sulfonamides as probes of tumor-associated carbonic anhydrase IX that inhibit isozyme IX-mediated acidification of hypoxic tumors. J Med Chem 2005;48:4834–41. 16033263 10.1021/jm0501073

[18] Svastová E, Huliková A, Rafajová M, et al Hypoxia activates the capacity of tumor-associated carbonic anhydrase IX to acidify extracellular pH. FEBS Lett 2004;577:439–45. 15556624 10.1016/j.febslet.2004.10.043

[19] Supuran CT, Scozzafava A, Ilies MA, et al Carbonic anhydrase inhibitors – Part 53. Synthesis of substituted-pyridinium derivatives of aromatic sulfonamides: the first non-polymeric membrane-impermeable inhibitors with selectivity for isozyme IV. Eur J Med Chem 1998;33:577–94.

[20] Grandane A, Belyakov S, Trapencieris P, Zalubovskis R. Facile synthesis of coumarin bioisosteres – 1,2-benzoxathiine 2,2-dioxides. Tetrahedron 2012;68:5541–6.

[21] Žalubovskis R. In a search for selective inhibitors of carbonic anhydrases: coumarin and its bioisosteres – synthesis and derivatization. Chem Heterocycl Compd 2015;51:607–12.

[22] Krinke D, Jahnke H-G, Pänke O, Robitzki AA. A microelectrode-based sensor for label-free *in vitro* detection of ischemic effects on cardiomyocytes. Biosens Bioelectron 2009;24:2798–803. 19285854 10.1016/j.bios.2009.02.006

[23] Dinischiotu A, Stanca L, Gradinaru D, et al Lipid peroxidation due to *in vitro* and *in vivo* exposure of biological samples to nanoparticles In: Armstrong D, Bharali DJ, eds. Oxidative stress and nanotechnology: methods and protocols. Totowa (NJ): Humana Press; 2013:155–64. 10.1007/978-1-62703-475-3_10 23740119

[24] Arndt S, Seebach J, Psathaki K, et al Bioelectrical impedance assay to monitor changes in cell shape during apoptosis. Biosens Bioelectron 2004;19:583–94. 14683642 10.1016/s0956-5663(03)00269-0

[25] Ivanov S, Liao SY, Ivanova A, et al Expression of hypoxia-inducible cell-surface transmembrane carbonic anhydrases in human cancer. Am J Pathol 2001;158:905–19. 11238039 10.1016/S0002-9440(10)64038-2 PMC1850340

[26] Supuran CT. Carbonic anhydrase inhibitors and activators for novel therapeutic applications. Future Med Chem 2011;3:1165–80. 21806379 10.4155/fmc.11.69

[27] Liu T, Laurell C, Selivanova G, et al Hypoxia induces p53-dependent transactivation and Fas/CD95-dependent apoptosis. Cell Death Differ 2006;14:411–21. 16917513 10.1038/sj.cdd.4402022

[28] Behn C, Araneda OF, Llanos AJ, et al Hypoxia-related lipid peroxidation: evidences, implications and approaches. Respir Physiol Neurobiol 2007;158:143–50. 17662674 10.1016/j.resp.2007.06.001

[29] Koumenis C, Alarcon R, Hammond E, et al Regulation of p53 by hypoxia: dissociation of transcriptional repression and apoptosis from p53-dependent transactivation. Mol Cell Biol 2001;21:1297–310. 11158315 10.1128/MCB.21.4.1297-1310.2001 PMC99582

[30] Supuran CT, Scozzafava A, Casini A. Carbonic anhydrase inhibitors. Med Res Rev 2003;23:146–89. 12500287 10.1002/med.10025

[31] Winum JY, Poulsen SA, Supuran CT. Therapeutic applications of glycosidic carbonic anhydrase inhibitors. Med Res Rev 2009;29:419–35. 19058143 10.1002/med.20141

[32] Carta F, Supuran CT, Scozzafava A. Sulfonamides and their isosters as carbonic anhydrase inhibitors. Future Med Chem 2014;6:1149–65. 25078135 10.4155/fmc.14.68

[33] Menchise V, De Simone G, Alterio V, et al Carbonic anhydrase inhibitors: stacking with Phe131 determines active site binding region of inhibitors as exemplified by the X-ray crystal structure of a membrane-impermeant antitumor sulfonamide complexed with isozyme II. J Med Chem 2005;48:5721–7. 16134940 10.1021/jm050333c

[34] Supuran CT. Carbonic anhydrases: novel therapeutic applications for inhibitors and activators. Nat Rev Drug Discov 2008;7:168–81. 18167490 10.1038/nrd2467

[35] Dubois L, Lieuwes NG, Maresca A, et al Imaging of CA IX with fluorescent labelled sulfonamides distinguishes hypoxic and (re)-oxygenated cells in a xenograft tumour model. Radiother Oncol 2009;92:423–8. 19616332 10.1016/j.radonc.2009.06.019

[36] Mansfield KD, Simon MC, Keith B. Hypoxic reduction in cellular glutathione levels requires mitochondrial reactive oxygen species. J Appl Physiol (1985) 2004;97:1358–66. 15180977 10.1152/japplphysiol.00449.2004

[37] Meyer AJ, Hell R. Glutathione homeostasis and redox-regulation by sulfhydryl groups. Photosynth Res 2005;86:435–57. 16315075 10.1007/s11120-005-8425-1

[38] Zhang H, Bosch-Marce M, Shimoda LA, et al Mitochondrial autophagy is an HIF-1-dependent adaptive metabolic response to hypoxia. J Biol Chem 2008;283:10892–903. 18281291 10.1074/jbc.M800102200 PMC2447655

[39] Lai MC, Chang CM, Sun HS. Hypoxia induces autophagy through translational up-regulation of lysosomal proteins in human colon cancer cells. PLoS One 2016;11:e0153627. 27078027 10.1371/journal.pone.0153627 PMC4831676

[40] Alterio V, Di Fiore A, D’Ambrosio K, et al Multiple binding modes of inhibitors to carbonic anhydrases: how to design specific drugs targeting 15 different isoforms? Chem Rev 2012;112:4421–68. 22607219 10.1021/cr200176r

[41] Tanc M, Carta F, Bozdag M, et al 7-Substituted-sulfocoumarins are isoform-selective, potent carbonic anhydrase II inhibitors. Bioorg Med Chem 2013;21:4502–10. 23769167 10.1016/j.bmc.2013.05.032

[42] Nocentini A, Ceruso M, Carta F, Supuran CT. 7-Aryl-triazolyl-substituted sulfocoumarins are potent, selective inhibitors of the tumor-associated carbonic anhydrase IX and XII. J Enzyme Inhib Med Chem 2016;31:1226–33. 26681367 10.3109/14756366.2015.1115401

[43] Grandane A, Tanc M, Di Cesare Mannelli L, et al 6-Substituted sulfocoumarins are selective carbonic anhydrase IX and XII inhibitors with significant cytotoxicity against colorectal cancer cells. J Med Chem 2015;58:3975–83. 25875209 10.1021/acs.jmedchem.5b00523

[44] Gu W, Zhao Y. Cellular electrical impedance spectroscopy: an emerging technology of microscale biosensors. Expert Rev Med Devices 2010;7:767–79. 21050088 10.1586/erd.10.47

[45] Hong J, Kandasamy K, Marimuthu M, et al Electrical cell-substrate impedance sensing as a non-invasive tool for cancer cell study. Analyst 2011;136:237–45. 20963234 10.1039/c0an00560f

[46] Abassi YA, Xi B, Zhang W, et al Kinetic cell-based morphological screening: prediction of mechanism of compound action and off-target effects. Chem Biol 2009;16:712–23. 19635408 10.1016/j.chembiol.2009.05.011 PMC2836189

[47] Zieseniss A. Hypoxia and the modulation of the actin cytoskeleton – emerging interrelations. Hypoxia (Auckl) 2014;2:11–21. 27774463 10.2147/HP.S53575 PMC5045051

