# Supplementary material for: Quantitative assessment of specific carbonic anhydrase inhibitors effect on hypoxic cells using electrical impedance assays
Source: J Enzyme Inhib Med Chem. 2017 Aug 7;32(1):1079–90. doi: 10.1080/14756366.2017.1355306 (PMC6010035; doi:10.1080/14756366.2017.1355306)
Supplement: IENZ_1355306_Supplemental_Material.pdf [file IENZ_A_1355306_SM3271.pdf]

Quantitative assessment of specific carbonic anhydrase inhibitors effect on hypoxic cells using electrical impedance assays

Luciana Stanica, Mihaela Gheorghiu, Miruna Stan, Cristina Polonschii, Sorin David, Dumitru Bratu, Anca Dinischiotu, Claudiu T. Supuran, Eugen Gheorghiu#

#*Corresponding author:* egheorghiu@biodyn.ro

The effect of hypoxia on cell viability, lipid peroxidation and p53 levels

Figure S1

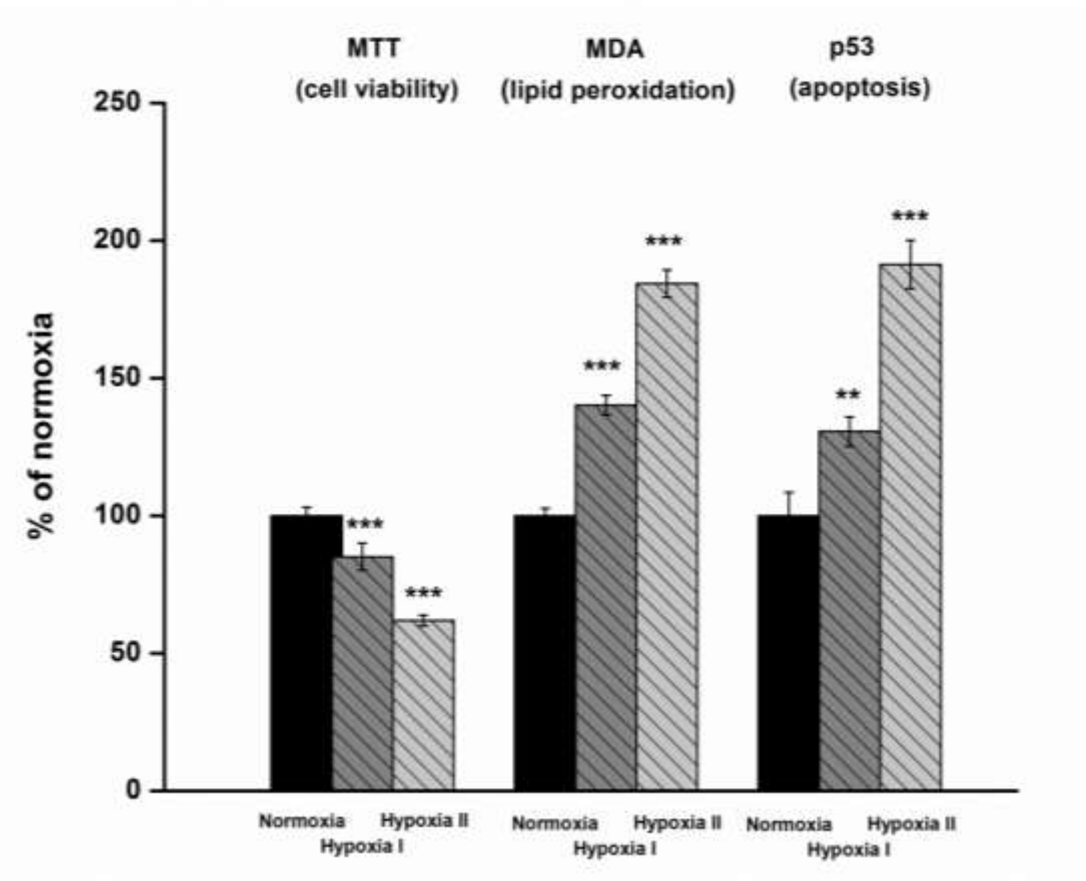

Cell viability, lipid peroxidation and p53 protein level in response to hypoxia. The results are represented as percentage of control (normoxia), considered 100%. Data are expressed as mean ± SD for n=6 (MTT), n=5 (MDA and p53 expression), ***p*<0.01 and ****p*<0.001 vs. normoxic conditions considered control.

Cell morphology, CA IX expression and CAI #1 binding

Figure S2

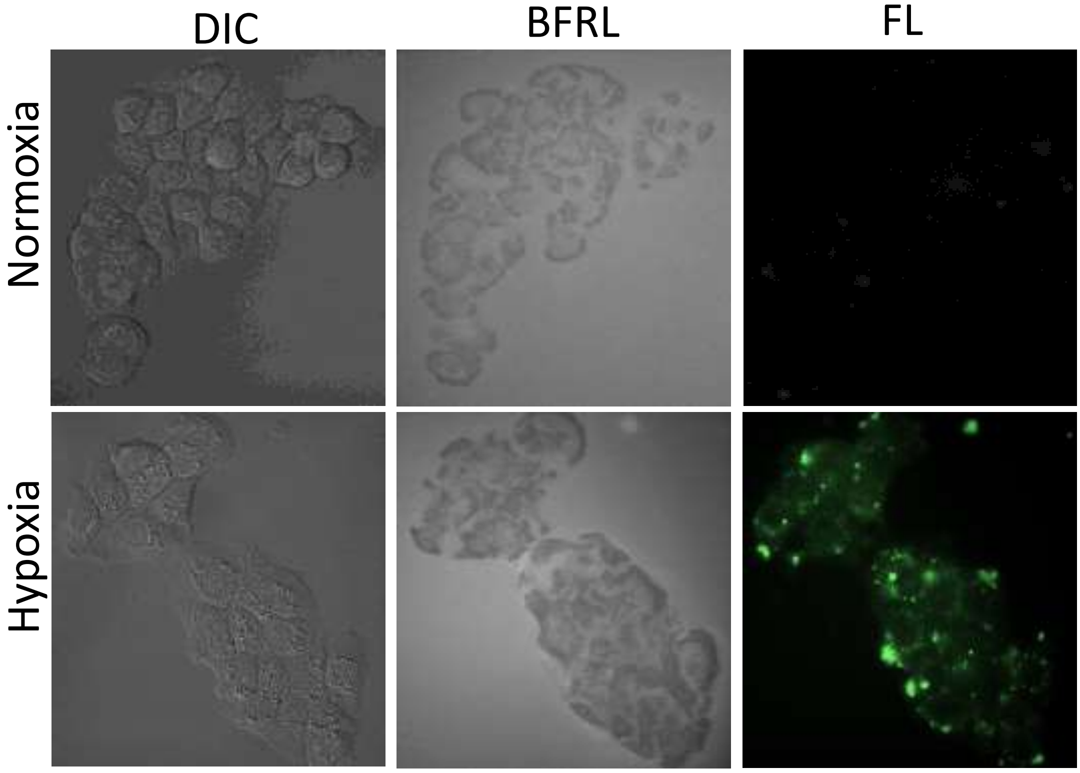

A

Microscopy images revealing morphology, adherence and structural changes for cells in normoxic and hypoxic conditions; DIC - Differential Interference Contrast, BFRL - Bright Field Reflected Light, FL - Green Fluorescence images corresponding to CA(IX)I #1 binding (false coloring).

Flatter, more stressed (i.e. more vacuoles present) cell appearance for hypoxic cells is evident in DIC and BFRL images. CA IX overexpression in hypoxic conditions and CAI #1 binding are evident in FL images.

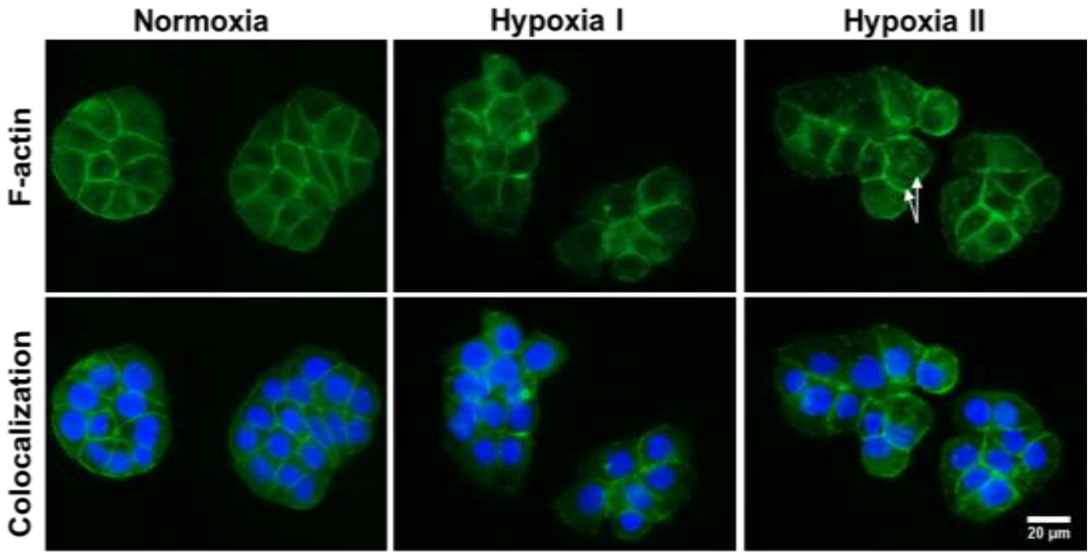

B

Actin cytoskeleton organization in response to hypoxia (green: F-actin labelled with phalloidin-FITC; blue: nuclei stained with DAPI). Note the stress fibres (arrows) in the cells following the second phase of hypoxia. Scale bar, 20 µm.

Equivalent circuit analysis

The experimental data obtained from the whole measured frequency domain, 100 Hz-100 kHz were fitted with an equivalent electrical circuit (inset Figure 1A) containing a constant phase element (CPE) which represents the cell monolayer-electrode interface and is characterized by two independent parameters: the amplitude, CPE_T and the distribution parameter CPE_P, ($Z_{CPE} = \frac{1}{CPE_{\_T} \cdot (i \cdot \omega)^{CPE_{\_P}}}$), a resistor (Rp) that defines the paracellular route in parallel with a capacitor (Cc) which describes the cellular monolayer and a resistor for the bulk solution (Rsol).

The values of the elements of the electrical circuit obtained after fitting were compared for normoxic cells, hypoxic cells and hypoxic cells incubated with different inhibitors to obtain complementary information regarding the bioeffects induced by these compounds on HT29 cells. Normalized data, $\frac{V(t)-V(0)}{V(0)} * 100$ are represented in % as mean ± SD for n=3 for all circuit parameters.

Figure S3

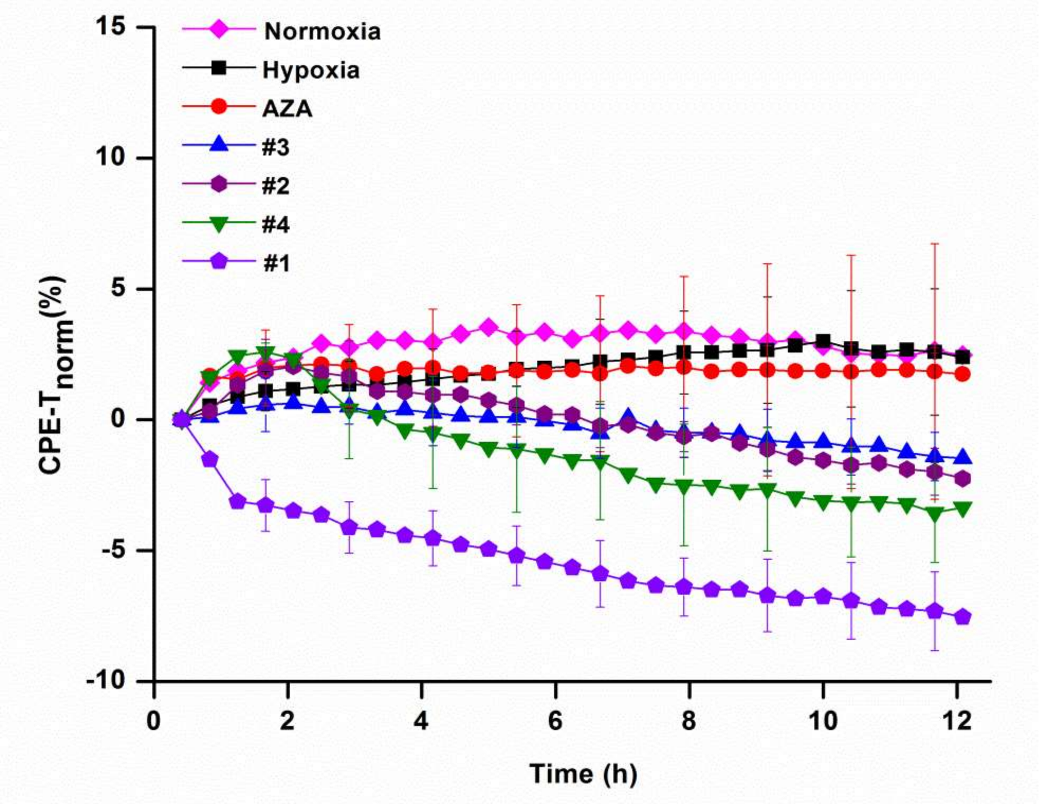

The dynamics of a characteristic equivalent circuit component CPE_$_T$ derived from complex fitting of impedance data during 12 h of hypoxia as function of model CAIs exposure (100 μM).

The pronounced drop in CPE_$_T$ values for CAI #1 suggests significant cell detachment and looser cell-surface contacts in response to efficient CA IX inhibition by this compound. A different interaction mechanism and a more reduced potency is highlighted for CAI #4: in the first couple of hours the dynamics is similar to control (normoxic conditions) whereas the following downward trend indicates looser cell-surface contacts in response to CA IX inhibition.

Figure S4

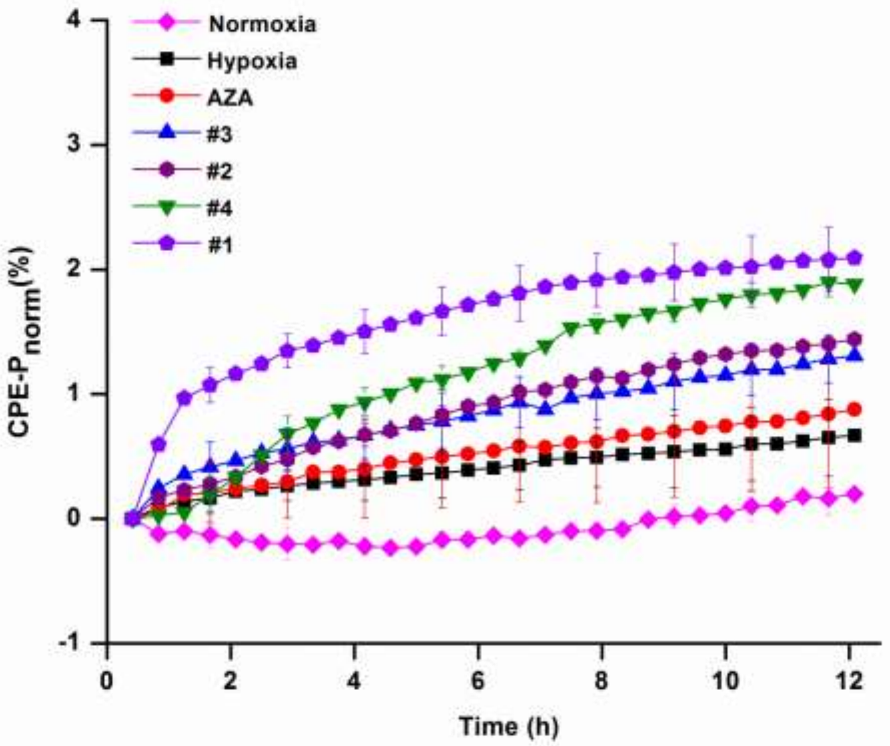

The dynamics of a characteristic equivalent circuit component $CPE_{\_P}$ derived from complex fitting of impedance data during 12 h of hypoxia as function of model CAIs exposure (100 µM).

CPE is used in the model to compensate for non-homogeneity in the system in conjunction with the rough/porous surface of the electrodes and inhomogeneous cell attachment. When the value of $CPE_{\_P}$ equals 1, the CPE behaves as an ideal capacitor, whereas when 0, the CPE behaves as a pure resistor. $CPE_{\_P}$ values between 0.9 and 1 have been derived from the fitting; more potent the inhibitor, the more pronounced the increase of the normalized $CPE_{\_P}$ values during exposure.

Figure S5

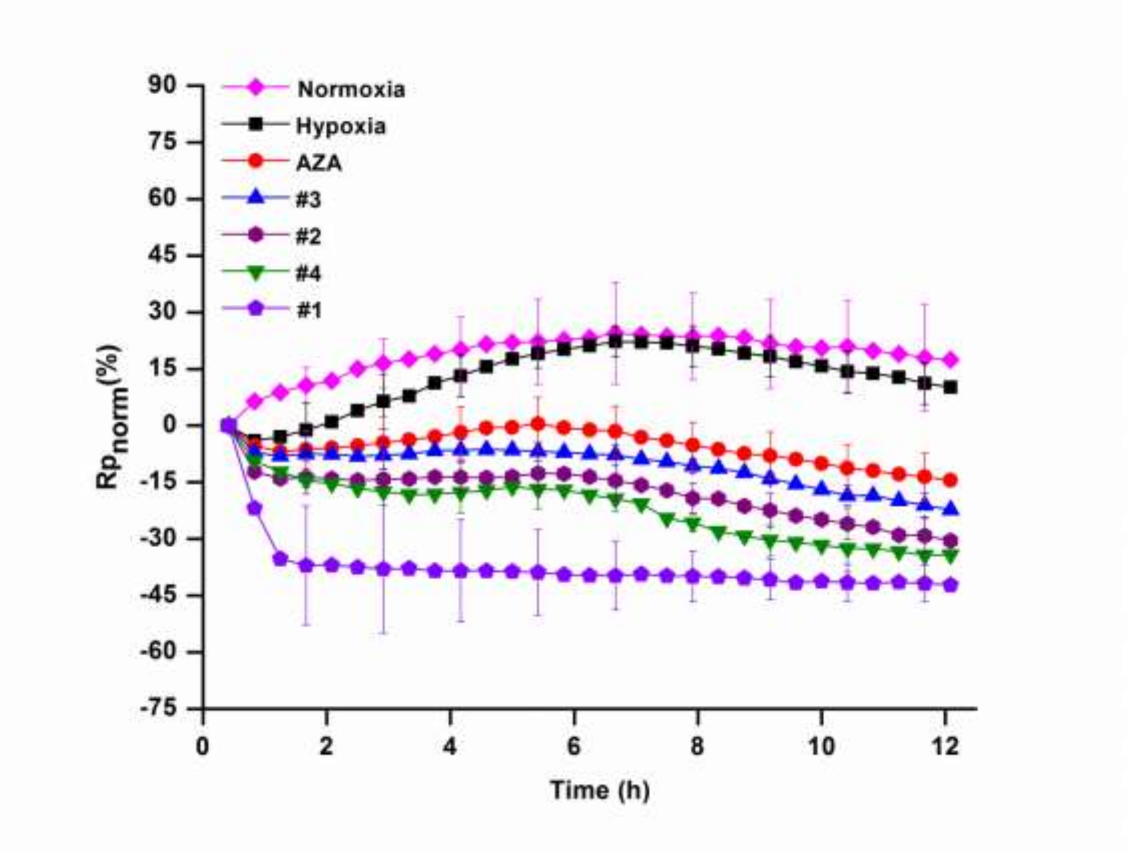

The dynamics of a characteristic equivalent circuit component Rp derived from complex fitting of impedance data during 12 h of hypoxia as function of model CAIs exposure (100 µM).

CAI #1>CAI #4>CAI #2>CAI #3>AZA effect on monolayer tightness can be inferred from Rp evolutions.

Figure S6

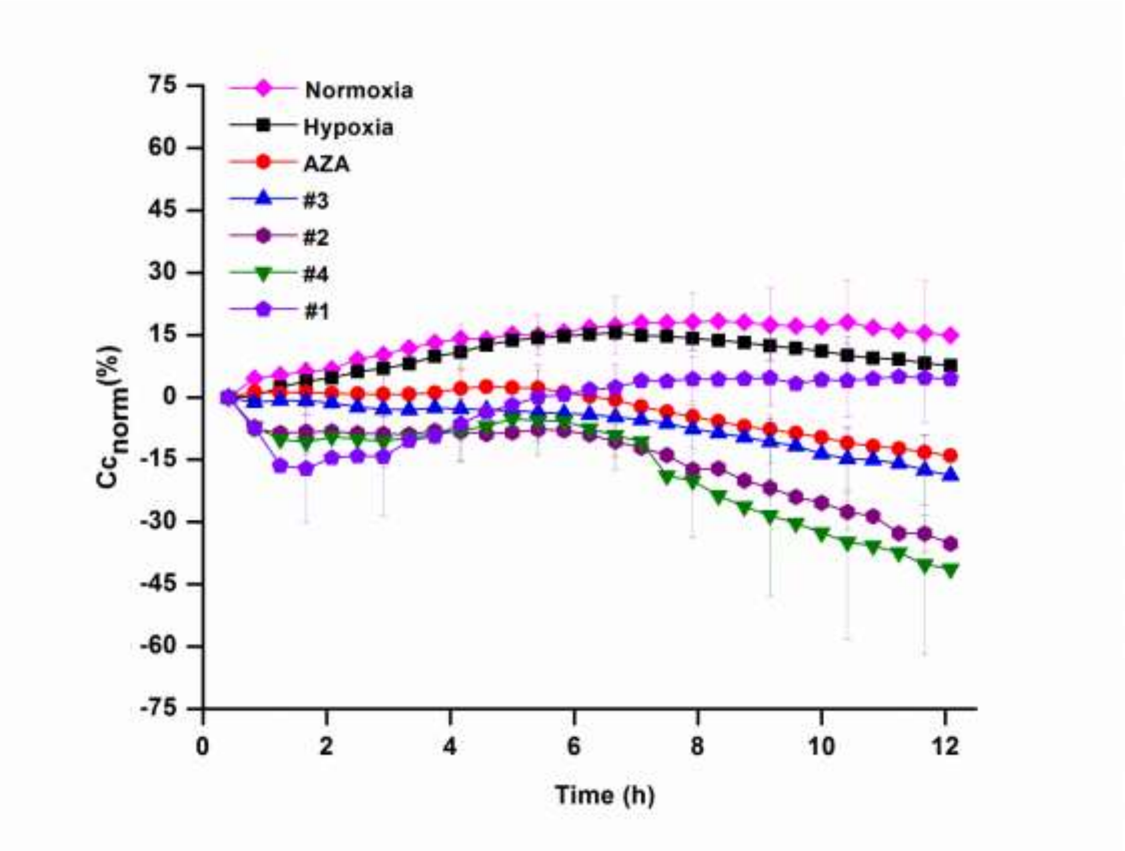

The dynamics of a characteristic equivalent circuit component Cc derived from complex fitting of impedance data during 12 h of hypoxia as function of model CAIs exposure (100 μM).

For improved readability and comparison with the dynamics of the other parameters, Cc values were represented as $\frac{Cc(0)-Cc(t)}{Cc(0)} * 100$. Distinct dynamics from control (normoxia) and hypoxia are obtained for all tested inhibitors indicating high sensitivity of the parameter for the related cellular effects. The changes become significant after 6 h of combined exposure to hypoxia and CAIs.
